# Monitoring Tacrolimus Trough Concentrations During the First Year After Kidney Transplantation: A National Retrospective Cohort Study

**DOI:** 10.3389/fphar.2020.566638

**Published:** 2020-11-20

**Authors:** Sarah S. Alghanem, Moetaza M. Soliman, Ali A. Alibrahim, Osama Gheith, Ahmed S. Kenawy, Abdelmoneim Awad

**Affiliations:** ^1^ Department of Pharmacy Practice, Kuwait University, Kuwait City, Kuwait; ^2^ Department of Pharmacy Practice, Faculty of Pharmacy, Mansoura University, Mansoura, Egypt; ^3^ Pharmacy Department, Manahi Al-Osaimi Health Centre, Ministry of Health, Kuwait City, Kuwait; ^4^ Nephrology Department, Hamed Al-Essa Organ Transplant Centre, Ministry of Health, Kuwait City, Kuwait; ^5^ Urology and Nephrology Centre, Mansoura University, Mansoura, Egypt; ^6^ Pharmacy Department, Hamed Al-Essa Organ Transplant Centre, Ministry of Health, Kuwait city, Kuwait

**Keywords:** tacrolimus, kidney transplantation, posttransplant diabetes, posttransplant hypertension, posttransplant dyslipidemia, calcinurin inhibitors

## Abstract

**Background:** There is a lack of data in the literature on the evaluation of tacrolimus (TAC) dosage regimen and monitoring after kidney transplantation (KT) in Kuwait. The aim of the present study was to evaluate TAC dosing in relation to the hospital protocol, the achievement of target TAC trough concentration (C_0_), the prevalence of TAC side effects (SEs), namely, posttransplant diabetes mellitus (PTDM), denovo hypertension (HTN), and dyslipidemia, and factors associated with the occurrence of these SEs among KT recipients.

**Methods:** A retrospective study was conducted among 298 KT recipients receiving TAC during the first year of PT. Descriptive and multivariate logistic regression analyses were used.

**Results:** The initial TAC dosing as per the local hospital protocol was prescribed for 28.2% of patients. The proportion of patients who had C_0_ levels within the target range increased from 31.5 to 60.3% during week 1 through week 52. Among patients who did not have HTN, DM, or dyslipidemia before using TAC, 78.6, 35.2, and 51.9% of them were prescribed antihypertensive, antidiabetic, and antilipidemic medications during the follow-up period. Age of ≥40 years was significantly associated with the development of *de novo* HTN, dyslipidemia, and PTDM (*p* < 0.05). High TAC trough concentration/daily dose (C_0_/D) ratio was significantly associated with the development of PTDM (*p* < 0.05).

**Conclusion:** Less than two-fifths of patients achieved target TAC C_0_ levels during the first month of PT. Side effects were more common in older patients. These findings warrant efforts to implement targeted multifaceted interventions to improve TAC prescribing and monitoring after KT.

## Introduction

Tacrolimus (TAC) is the cornerstone immunosuppressive (IS) agent after solid-organ transplantation. Its use as a medicine is complicated by having a narrow therapeutic index and varying intra- and interindividual variabilities (Staatz and Tett, 2004). Therefore, therapeutic drug monitoring (TDM) is used to individualize TAC dosages and reduce the risks of toxicity and rejection, with published guidelines for that purpose (Jusko et al., 1995; Wallemacq et al., 2009; Brunet et al., 2019). The area-under-the-concentration (AUC) vs. time curve was reported as the exposure metrics best associated with TAC clinical outcomes and was suggested to be the preferable measure of drug exposure (Brunet et al., 2019). However, no prospective study has been conducted in adult or pediatric transplantation to investigate the potential benefits on clinical outcomes of AUC monitoring over trough concentration (C_0_) monitoring. It was reported that AUC_0–12_ correlated better with C_12_ than C_0_ for twice daily TAC. However, the authors concluded that C_0_ can be a correct proxy of the overall exposure if blood sampling is correctly timed (Marquet et al., 2018). Therefore, whole-blood C_0_ is still used to adjust TAC dosing in most transplant centers until more evidence emerges.

The clinical use of TAC is associated with the risk of nephrotoxicity, neurotoxicity, hypertension (HTN), dyslipidemia, and posttransplant diabetes mellitus (PTDM). Only a few studies have investigated the relationship between TAC exposure and the risk of toxicity (Kershner and Fitzsimmons, 1996; Bottiger et al., 1999). However, these studies included a small sample size (14–92 patients) and used doses (0.1–0.4 mg/kg/day) and target TAC levels (5–40 ng/ml) higher than those used in recent years. The conclusion from these studies was that TAC monitoring is beneficial, and the target level should be kept less than 20 ng/ml and preferably less than 10 ng/ml to reduce the risk of side effects (SEs) (Kershner and Fitzsimmons, 1996; Bottiger et al., 1999).

In Kuwait, the number of patients with kidney transplantation (KT) is increasing with 20 living donors in the early 1990s to 77 patients (47 living and 30 deceased donors) in 2015. Few studies were published in the Middle East and North Africa (MENA) region to compare TAC-based vs. cyclosporine-based regimens and associated outcomes (El-Agroudy et al., 2008; Alghamdi et al., 2011), examined TAC dose requirements among their population (Mohsin et al., 2005), and assess factors associated with the development of PTDM following KT (Al-Ghareeb et al., 2012; Abdulrahman et al., 2018). However, there are no studies in the literature that evaluated TAC dosage regimen, monitoring, and associated outcomes after KT in Kuwait. Therefore, this study was designed to assess the physicians’ adherence to hospital protocol regarding the dosing and monitoring of TAC among KT recipients, and the achievement of target TAC trough concentration within the first year of posttransplantation (PT). Also, it identified the prevalence of TAC SEs, namely, PTDM, HTN, and dyslipidemia during the first year of PT and the factors associated with the occurrence of these SEs.

## Materials and Methods

### Study Design and Patient Population

This was a retrospective observational cohort study that had recruited KT patients receiving TAC-based primary IS regimen and followed up in Hamed Al-Essa Organ Transplant Centre, Ministry of Health, Kuwait, which is the only center for KT in Kuwait. TAC was introduced in Kuwait since 1992, and hence, data were included from 1992 until 2015. Patients on TAC, those aged 18 years old and older, with immediate, slow, or delayed graft function, and those with first, second, or multiple transplants were included. The exclusion criteria were as follows: patients who were lost to follow-up or death during the first 3 months after transplantation and those with unknown dates of transplantation recorded in the database. Ethical approval was obtained from the “Human Ethical Committee, Ministry of Health, Kuwait”.

A local hospital database was created in 2009 and included all patients since 1992 who were transplanted and followed up in the transplant center. The total number of patients in the database was 2,579 patients; of whom, 548 patients were prescribed TAC as a primary IS therapy. After excluding patients who did not meet the inclusion criteria or had their transplant outside Kuwait and no data were available for the first 3 months PT, 298 patients remained and included in the study. All these patients were transplanted during the period from 2001 to 2015.

### Study Setting

The Nephrology Department of the Hamed Al-Essa Organ Transplant Centre works in harmony with departments of transplant surgery, immunology, laboratories, and other subsidiary facilities in Ibn Sina Hospital. This program is the largest single-center program in the MENA region and performs the biggest number/million population transplants per year. The center performs all types of pre- and posttransplant procedures, treatment, and rehabilitation.

### Tacrolimus Dose and Monitoring

The local hospital protocol consisted of doses of antithymocyte globulin (Sanofi US, Bridgewater, NJ, United States) for high-risk patients or two doses of IL-2 receptor blocker (basiliximab; Novartis, Inc., Switzerland) for low-risk patients. TAC is initiated on day 1 or day 2 PT with a dose of 0.15 mg/kg per day for years 2001 to 2007 and 0.1 mg/kg per day from 2008 onwards administered in two divided doses for receipts with immediate graft function and 0.05 mg/kg per day for those with slow or delayed graft function for both years. Subsequent doses were adjusted according to whole-blood concentrations to achieve target C_0_ levels of 10–15 ng/ml (week 1), 7–10 ng/ml (week 2–12), and 5–7 ng/ml (week 13–52) for years 2001 to 2007. In 2008, the protocol was updated, and subsequent doses are adjusted according to blood concentrations to achieve target C_0_ levels of 10–12 ng/ml (week 1and 2), 8–10 ng/ml (week 3–12), and 5–8 ng/ml (week 13–52). For patients with delayed graft function, subsequent doses are adjusted according to whole-blood concentrations to achieve target C_0Wb_ levels of 5–7 ng/ml (week 1–2), then adjusted to achieve target C_0_ levels as mentioned above. Patients were followed up twice per week during first month, once weekly during second month, then bimonthly for 2–3 months, and then monthly till the end of the first year.

### Data Collection

Data were collected from the local hospital database using a standardized data collection form. The data collection form is composed of four sections. The first section recorded the patients’ demographic characteristics (age, gender, weight, and height). Section two included information about graft function immediate, slow, or delayed graft function, whether it was first or multiple transplants, IS regimen, and biochemical parameters (serum creatinine (Scr), potassium, and hematocrit). Glomerular filtration rate was calculated by the Chronic Kidney Disease Epidemiology (CKD-EPI) equation (Levey et al., 2009). The third section consisted of information about TAC dosage regimen and concomitant medications. The information collected related to TAC dosing and monitoring included i) TAC dosage regimen, ii) measured TAC C_0Wb_ level, and iii) modification in dose by the physician when applicable at the start and at weeks 1, 2, 3, 4, 8, 12, 24, and 52. Finally, the fourth section included information about comorbidities at the time of transplant, including diagnosis with diabetes mellitus (DM), HTN, and dyslipidemia. Also, it includes information about the initiation of medications for these conditions during the follow-up.

### TAC Measurement Assay

TAC immunoassay was introduced at the transplant center laboratory since 2000. TAC whole-blood concentrations were measured by the microparticle enzyme immunoassay method based on the Abbott IMx analyzer. The limit of detection for this assay was 0.5 ng/ml.

### Statistical Analysis

Descriptive and logistic regression analyses were conducted using Stata 10.1 software (Stata Corp., College Station, TX, United States). Normality test was conducted for continuous data using the Anderson Darling test. The results were presented as percentages (95% confidence intervals, CI), means (standard deviation - SD) for normally distributed data, and medians (interquartile range, IQR) for skewed data. Adherence to the local hospital TAC dosage protocol and monitoring among KT recipients were assessed by comparing the actual administered dose in milligrams/kilogram per day with the recommended dose in the protocol. Proportions of patients who achieved target TAC C_0_ level or out of the range were determined at different follow-up times after transplantation. Follow-up data were presented over eight times during 1 year after transplantation.

Univariate and multivariate logistic regression models were used to identify factors associated with the development of SEs to TAC. For TAC-associated SEs analysis, patients without baseline diagnosis of clinical conditions (DM, HTN, and dyslipidemia) were included only to differentiate between clinical conditions and SEs. In the present study, assessment of TAC-associated SEs was performed after 3 months of transplantation because during the first 3 months after transplant, patients are usually unstable and might suffer from transient HTN and hyperglycemia as SEs from high corticosteroid doses to treat rejection. Therefore, guidelines recommend assessing immunosuppressant SEs after 3 months of transplant, so patients are more stable. A guideline with a clear definition to evaluate immunosuppressant-associated SEs was published for PTDM (Sharif et al., 2014; Torres et al., 2018). However, there was no clear clinical diagnostic guideline for HTN- or dyslipidemia-induced by TAC. Therefore, these SEs as dependent variables were defined by the initiation of medication therapy for the treatment of HTN, DM, or dyslipidemia and its regular use after the third month and up to the study follow-up time (Sharif et al., 2014; Torres et al., 2018). Variables that showed a significance level of *p* < 0.05 in the univariate analysis were included in the multivariate model. Covariates included baseline demographics (age, gender, body mass index (BMI)), TAC C_0_ level to TAC dose (C_0_/D) ratio, TAC C_0_ level, and medication interactions that increase TAC level. Age was categorized to three groups (18–39, 40–59, and ≥60 years). TAC C_0_/D ratio was categorized to three groups (<1, 1–2, and >2 [ng/ml]/mg). TAC C_0_ level was categorized to three groups (<8, 8–11, and >11 ng/ml). The results for logistic models were presented as odds ratios (OR) (95% CI). A significant level was set at ≤0.05.

## Results

### Demographic and Clinical Characteristics of Patients

Table 1 shows the patients’ characteristics. Of the 298 patients, 161 (54.0%) were males and 137 (46.0%) were females. Their mean (SD) age and BMI were 46.5 (13.8) years and 28.2 (6.1) kg/m^2^, respectively. More than one-third (37.2%; 95% CI, 31.8–43.0) of the patients were obese. Two-hundred fifty-four patients (85.2%; 95% CI, 80.6–89.0) had a transplant for the first time.

**TABLE 1:** Demographic and clinical characteristics of patients (n = 298).

	Total (n = 298)
Gender (n, %)	
Male	161 (54.0)
Female	137 (46.0)
Age category (n, %)	
18–39 years	99 (33.2)
40–59 years	147 (49.3)
≥60 years	52 (17.5)
Height (cm) (mean (SD))	164.2 (9.3)
Weight (kg) (mean (SD))	76.0 (17.8)
BMI (kg/m^2^) (mean (SD))	28.2 (6.1)
BMI category (n, %)	
Underweight (< 18.5)	15 (5.0)
Normal (18.5–24.9)	80 (26.9)
Overweight (25–29.9)	92 (30.9)
Obese (≥ 30)	111 (37.2)
Graft function (n, %)	
Immediate	215 (72.2)
Slow	65 (21.8)
Delayed	18 (6.0)
Transplant	
First time	254 (85.2)
Second time	42 (14.1)
Third time	2 (0.7)

BMI, body mass index. Height was missing in 84 patients, and hence, the median height for relevant gender was used to estimate body max index.

The comorbidities among the patients at the time of transplant were HTN (n = 242; 81.2%; 95% CI, 76.2–85.4), DM (n = 102; 34.2%; 95% CI, 28.9–40.0), dyslipidemia (n = 66; 22.1%; 95% CI, 17.7–27.4), ischemic heart disease (n = 32; 10.7%; 95% CI, 7.6–15.0), epilepsy (n = 11; 3.7; 95% CI, 2.0–6.7), atrial fibrillation (n = 10; 3.4%; 95%CI: 1.7–6.3), and heart failure (n = 1; 0.3%; 95% CI, 0.02–2.2).

### Patients’ Biochemical Data and Renal Function at Baseline and During the First Year of Transplantation

Table 2 presents the patients’ biochemical data at baseline and during the first year of transplantation. The Scr levels at the start of TAC administration were high with a median (IQR) value of 277 (171–440) µmol/L, which continued to be slightly high at 121 (90–188) µmol/L in the first week and decreased to 101 (85–133) µmol/L by the end of follow-up time. Renal function was improving over time with a median (IQR) GFR of 54 ml/min/1.73 m^2^ (32–76) at week 1 and reached 66 ml/min/1.73 m^2^ (51–82) by week 52. The mean (SD) level for serum potassium was within the normal range during the whole year, ranging from 4 (0.5) to 4.7 (0.6) mmol/L. Hematocrit fractions on the other hand were low at baseline and during week 1 with a mean (SD) value of 0.3 (0.0) L/L, which improved to 0.4 (0.0) L/L starting from week four till 52.

**TABLE 2:** Patients’ biochemical data at baseline and during 1 year after transplantation (n = 298).

	Baseline	Week 1	Week 2	Week 3	Week 4	Week 8	Week 12	Week 24	Week 52
Serum creatinine (µmol/L) median (IQR)	278 (172–437)	121 (90–188)	119 (91–168)	112 (85–151)	111 (87–146)	107 (86–134)	103 (85–133)	106 (86–135)	101 (85–133)
Glomular filtration rate (ml/min/1.73 m^2^) median (IQR)	—	54 (32–76)	54 (36–76)	60 (41–80)	60 (43–77)	61 (48–81)	64 (50–80)	63 (48–79)	66 (51–82)
Potassium level (mmol/L) mean (SD)	4.3 (0.6)	4.0 (0.5)	4.6 (0.6)	4.7 (0.6)	4.6 (0.6)	4.5 (0.5)	4.4 (0.4)	4.3 (0.5)	4.3 (0.5)
Hematocrit (L/L) mean (SD)	0.29 (0.05)	0.29 (0.05)	0.30 (0.05)	0.33 (0.05)	0.35 (0.05)	0.40 (0.06)	0.40 (0.05)	0.40 (0.05)	0.41 (0.05)

Glomerular filtration rate calculated by the chronic kidney diseases epidemiology (CKD−EPI) equation.

### Medication Use at Baseline and During the First Year After Transplantation

IS regimen used over the first year of follow-up is presented in Table 3 with most of the patients on prednisolone and mycophenolate acid (MPA) regimen (86.2%). Most of the patients (n = 278; 93.3%; 95% CI, 89.7–95.8) were on prednisolone and MPA regimen at the time of TAC initiation. A total of 242 patients (81.2%) were on antihypertensive, 102 (34.2%) were on antidiabetic, and 66 (22.1%) were on antilipidemic medications before transplantation as shown in Table 3. Drugs that interact with TAC and influence its trough level were coadministered in 187 patients (62.7%), 184 (98.4%) of them were on medications that can increase TAC concentration including proton pump inhibitors (PPIs), and three patients were on medications that decrease TAC concentration (antiepileptic (carbamazepine)).

**TABLE 3:** Number (%) of patients using immunosuppressive, antihypertensive, antidiabetic, and antilipdemic medications at baseline and during 1 year after transplantation (n = 298).

	Baseline	W1	W2	W3	W4	W8	W12	W24	W52
Immunosuppressive regimen									
Prednisolone	9 (3.0)	16 (5.4)	30 (10.1)	28 (9.4)	35 (11.7)	27 (9.1)	30 (10.1)	28 (9.4)	29 (9.7)
Prednisolone + mycophenolic acid	278 (93.3)	271 (90.9)	258 (86.6)	257 (86.2)	249 (83.6)	257 (86.2)	254 (85.2)	255 (85.6)	254 (85.2)
Other	11 (3.7)	11 (3.7)	10 (3.4)	13 (4.4)	14 (4.7)	14 (4.7)	14 (4.7)	15 (5.0)	15 (5.0)
Number of patients on medications for hypertension, diabetes, and dyslipidemia, n (%)									
On antihypertensive	242 (81.2)	277 (93.0)	277 (93.0)	280 (94.0)	280 (94.0)	284 (95.3)	285 (95.6)	285 (95.6)	286 (96.0)
On antidiabetic	102 (34.2)	117 (39.3)	124 (41.6)	134 (45.0)	137 (46.0)	153 (51.3)	162 (54.4)	167 (56.0)	171 (57.4)
On antilipidemic	66 (22.1)	110 (36.9)	125 (41.9)	137 (46.0)	140 (47.0)	158 (53.0)	168 (56.4)	180 (60.4)	187 (62.8)
Medications interact with TAC									
Increase TAC level (PPIs)	184 (61.7)	196 (65.8)	198 (66.4)	195 (65.4)	193 (64.8)	185 (62.1)	189 (63.4)	191 (64.1)	181 (60.7)
Decrease TAC level (carbamzepine)	3 (1.0)	4 (1.3)	3 (1.0)	2 (0.7)	2 (0.7)	2 (0.7)	3 (1.0)	3 (1.0)	4 (1.3)
No interacting agent	111 (37.2)	98 (32.9)	97 (32.6)	101 (33.9)	103 (34.6)	111 (37.2)	106 (35.6)	104 (34.9)	113 (37.9)

### Tacrolimus Dosage and Monitoring Protocol

The median (IQR) starting dose for TAC was 5.0 (4.0–7.5) mg/day. The recommended initial dose of TAC as per the hospital protocol was prescribed in about one-third (n = 84; 28.2%; 95% CI, 23.2–33.7) of patients; of whom, 38 (45.2%) were with delayed or slow graft function and 46 (54.8%) with immediate graft function. More than half (n = 169; 56.7%; 95% CI, 50.9–62.4) of patients received lower doses; of whom, 145 (85.8%) had immediate graft function and 24 (14.2%) had slow or delayed graft function. Forty-five patients (15.1%; 95% CI, 11.3–19.8) received higher doses; of whom, 24 (53.3%) had immediate graft functions and 21 (46.7%) had slow or delayed graft function.

The first TAC C_0_ level was obtained according to the hospital protocol (2–3 days after initiation) in 81 patients (27.2%; 95% CI, 22.3–32.7). Of these patients, only 11 (13.6%; 95% CI, 7.3–23.4) had achieved the target TAC level, whereas 60 (74.1%; 95% CI, 62.9–83.0) and 10 (12.3%; 95% CI, 6.4–22.0) were below and above target levels, respectively. The median (IQR) of the first TAC trough level and the C_0_/D ratio of TAC were 5.7 (3.3–8.6) ng/ml and 1.0 (0.6–1.6) [ng/ml]/mg, respectively.

### Tacrolimus Trough Concentration Monitoring During the First Year After Transplantation

**FIGURE 1:**
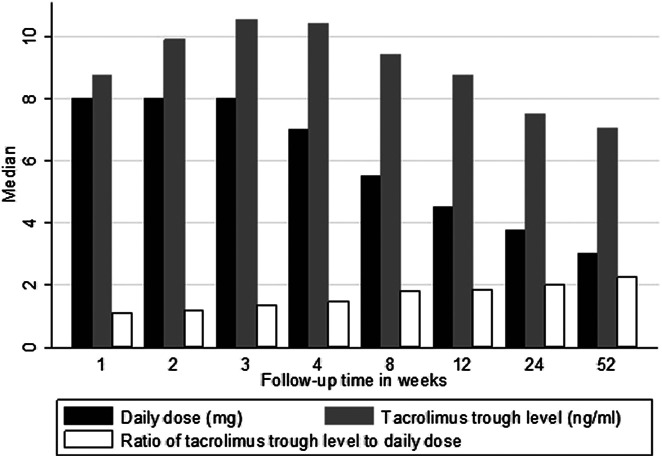
Median tacrolimus dose, trough levels, and C_0_/D ratio over 1 year of follow-up. Key: tacrolimus daily dose (mg/day); tacrolimus trough level (ng/ml); tacrolimus C_0_/D ratio [ng/ml]/mg.

The daily dose of TAC was decreasing over time as shown in Figure 1. The median (IQR) dose decreased from 8.0 (5.0–10.0) mg/day in the first week to 3.0 (2.0–4.5) mg/day in week 52. In total, 2093 TAC C_0_ levels were available for analysis during first year of follow-up. The median (IQR) TAC C_0_ level decreased from 8.8 (6.2–11.0) ng/ml in the first week to 7.0 (5.2–8.4) ng/ml in week 52 (Figure 1). Moreover, the median (IQR) TAC C_0_/D ratio increased from 1.1 (0.7–1.6) [ng/ml]/mg in the first week to 2.3 (1.5–3.1) [ng/ml]/mg in week 52 (Figure 1).

**FIGURE 2:**
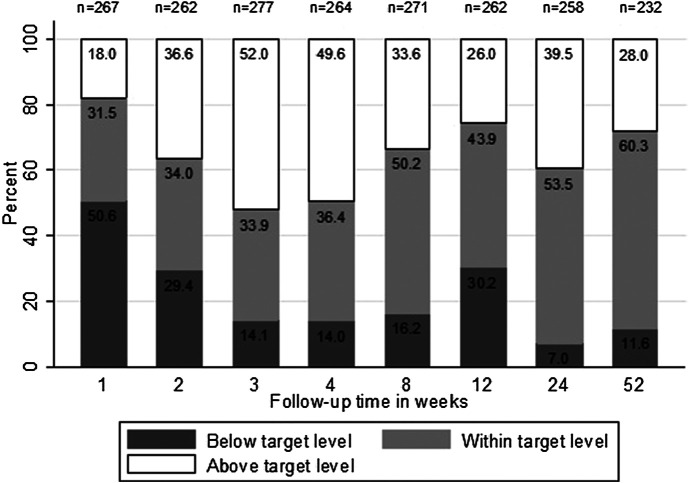
Percentage of patients achieving tacrolimus target trough concentration and those out of the range over 1 year of follow-up. Total number of patients with available tacrolimus trough level is presented on the top of each bar.

In the first week, of 267 patients, 84 (31.5%; 95% CI, 26.0–37.5) had C_0_ levels within the target range, whereas 89 out of 262 patients (34.0%; 95% CI, 28.4–40.1) were within the target range by the second week. There was an improvement in the achievement of target C_0_ levels over time where 140 of 232 patients (60.3%; 95% CI, 53.7–66.6) were within the target levels by the end of the 1-year follow-up (Figure 2). The highest proportion of patients (n = 144 of 277, 52.0%; 95% CI, 46.0–58.0) who had target levels above the range was seen in week 3. In total, there were 892 of 2093 (42.6%; 95% CI, 40.5–44.7) occasions where TAC C_0_ levels were within the target range during the entire year of follow-up.

Within each follow-up interval, following the initial TAC level monitoring, decisions were taken to modify the dose in 689 occasions throughout the first year after transplantation. The modifications were either reducing the dose as reported in 493 occasions (71.6%; 95% CI, 68.0–74.9) or increasing the dose as reported in 196 occasions (28.4%; 95% CI, 25.1–32.0).

### Tacrolimus Side Effects and Factors Associated With the Occurrence of These Side Effects

Of 56 patients who did not have HTN at baseline, 44 (78.6%; 95% CI, 65.2–88.0) had developed HTN and used antihypertensive medications (Table 3). From the 196 nondiabetic patients at baseline, 69 (35.2%; 95% CI, 28.6–42.4) had developed PTDM and used antidiabetic medications. Finally, for patients not having dyslipidemia at baseline (233 patients), 121 (51.9%; 95% CI, 45.3–58.5) have used antilipidemic agents.

The results of the univariate analysis for factors associated with TAC SEs are presented in the Supplementary Material (**Supplementary Table S1**). Table 4 presents the results of the multivariate analysis for factors associated with TAC SEs. The occurrence of HTN, PTDM, and dyslipidemia while receiving TAC was found to be more prevalent among patients aged 40–59 years and those of ≥60 years compared with those aged 18–39 years (*p* < 0.05). A higher risk was more common among those aged ≥60 years. Female patients were significantly less likely to develop HTN (OR, 0.60; 95% CI, 0.40–0.88) compared with males (*p* = 0.01), while a nonsignificant difference in risk was found between males and females for developing PTDM (*p* = 0.09).

**TABLE 4:** Factors associated with TAC side effects.

Characteristics	Developed hypertension after initiation of TAC and on antihypertensive medication(s) (n = 44)		Developed diabetes after initiation of TAC and on antidiabetic medication(s) (n = 69)		Developed dyslipidemia after initiation of TAC and on antilipidemic medication(s) (n = 121)	
	OR [95% CI]	*p* value	OR [95% CI]	*p* value	OR [95% CI]	*p*-value
Age (years)						
18–39	Reference		Reference		Reference	
40–59	1.71 [1.14–2.56]	0.01	1.98 [1.50–2.61]	0.00	2.00 [1.63–2.45]	0.00
≥60	2.07 [1.00–4.27]	0.05	2.20 [1.47–3.28]	0.00	3.33 [2.51–4.41]	0.00
Gender						
Male	Reference		Reference		Reference	
Female	0.60 [0.40–0.88]	0.01	1.24 [0.97–1.59]	0.09	—	—
BMI (Kg/m^2^)						
Underweight/normal	Reference		Reference		Reference	
Overweight/obese	—	—	2.03 [1.54–2.68]	0.00	1.29 [1.06–1.56]	0.01
TAC C_0_/D ratio [ng/ml]/mg						
<1	Reference		Reference		Reference	
1–2	—	—	1.64 [1.15–2.35]	0.01	—	—
>2	—	—	2.08 [1.47–2.95]	0.00	—	—
TAC C_0_ [ng/ml]						
<8	Reference —	—	Reference —	—	Reference —	—
8–11	—	—	—	—	0.64 [0.51–0.80]	0.00
>11	—	—	—	—	0.57 [0.46–0.70]	0.00
Use of medications that increase TAC level						
No	Reference		Reference		Reference	
Yes	—	—	—	—	—	—

OR, odds ratios; CI, confidence intervals; TAC, Tacrolimus; C0/D, tacrolimus concentration/dose; BMI, Body mass index; C0, tacrolimus trough concentration.

Patients who were overweight and obese were significantly more likely to develop PTDM (OR, 2.03; 95% CI, 1.54–2.68) and dyslipidemia (OR, 1.29; 95% CI, 1.06–1.56). Patients with a higher TAC C_0_/D ratio from 1 to 2 [ng/ml]/mg (OR, 1.64; 95% CI, 1.15–2.35) and >2 [ng/ml]/mg (OR, 2.08; 95% CI, 1.47–2.95) were more likely to develop PTDM compared with those with TAC C_0_/D ratio of <1 [ng/ml]/mg. Higher TAC C_0_/D ratio showed a nonsignificant effect on the risk of developing HTN and dyslipidemia (*p* > 0.05). Patients with a higher TAC C_0_ showed a lower risk of developing dyslipidemia but not HTN or PTDM. Receiving a medication that increases the level of TAC showed a nonsignificant effect on the risk of developing HTN, PTDM, and dyslipidemia.

## Discussion

The present findings revealed that only 28.2% of patients received the recommended initial dose of TAC as per the local hospital protocol, and the majority of first TAC levels (72.8%) were measured earlier than 2–3 days. Achievement of target TAC C_0_ level was variable during the first year with 42.6% of levels was within target during the first year after transplantation. The prevalence of TAC SEs among those who did not have HTN, DM, or dyslipidemia before using TAC was high with 78.6%, 35.2%, and 51.9% of them were prescribed antihypertensive, antidiabetic, and antilipidemic medications during the follow-up period, respectively. The present findings provide a useful baseline quantitative data set that will assist in the assessment of current TAC prescribing and monitoring patterns that should be utilized by the hospital authorities to design future targeted multifaceted interventions. Also, these results allow for crucial comparative work with existing and future studies in the MENA region and worldwide.

TAC dose modifications were more frequent during the first four weeks of PT and peak in week 4. These modifications were subsequently reduced as time after transplant increased and reached its lowest by week 52. The frequent dose modifications that occurred during the first 4 weeks might reflect frequent monitoring and adjustment of doses before reaching steady state as well as patient instability and fluid shifts during the first month after the transplant. These frequent modifications in doses would lead to delay in the early achievement of safe and effective TAC levels and can increase the risk of early acute rejection (Staatz et al., 2001; Borobia et al., 2009).

The current results showed that the achievement of target TAC C_0_ level was variable during the first year after transplantation and low as only 60.3% of patients were within the target levels by the end of the first year of follow-up. These results are in agreement with the subanalysis of the Symphony study, a large, multicenter, clinical trial, which described the challenges in achieving target IS concentration where 63.9% of patients had TAC levels within the target range at week 52 (Ekberg et al., 2009). Possible reasons for the difficulty to keep levels within the target range could be physicians’ nonadherence to the protocol, patients’ nonadherence to their therapy, and the high intrapatient variability in TAC PKs. In a recent study conducted in Kuwait, 60% of 120 KT recipients reported being adherent to their IS medications, which highlights the need for interventions to increase medication adherence among these patients (Kenawy et al., 2019). High variability in TAC level during the first year and throughout 5 years after transplantation was found to be associated with poor allograft outcomes, including late acute rejection, transplant glomerulopathy, and allograft survival (Sapir-Pichhadze et al., 2014; O’Regan et al., 2016). Therefore, it is worth finding an approach to achieve target concentration early and reduce variabilities such as the use of Bayesian forecasting tool to individualize TAC dosing, which was reported as a useful approach for patients to achieve target TAC level (Størset et al., 2015). Moreover, the within-patient variability should be considered as a potential biomarker of treatment outcome in KT (Borra et al., 2010). The coefficient of variation of TAC C_0_ beyond 6 months after transplantation was found to be a more relevant biomarker of TAC toxicity and IS efficacy than the sole C_0_ (Rodrigo et al., 2016; Vanhove et al., 2016). Patients with high-exposure variability were found to be at higher risk of developing histologic kidney lesions, graft loss, and of poorer long-term outcomes (Borra et al., 2010; Sapir-Pichhadze et al., 2014; O’Regan et al., 2016; Rodrigo et al., 2016; Shuker et al., 2016; Vanhove et al., 2016).

In the current study, the C_0_/D ratio was used as a surrogate marker of TAC clearance or metabolism speed, which was shown to be associated with graft outcome (Jouve et al., 2019; Jouve et al., 2020; Van Gelder et al., 2020). The present findings revealed that the C_0_/D ratio was increasing over the follow-up period even with decreasing doses. This could reflect the decrease in TAC clearance (CL) that may increase TAC bioavailability (Staatz and Tett, 2004). Another possible explanation is the use of corticosteroids among the majority of patients. It has been found that reduction in the dose of corticosteroids, from the early PT months to 1 year after transplantation, contributes to a decrease in TAC CL, and TAC doses should be reduced to maintain therapeutic concentrations (Undre and Schafer, 1998; Hesselink et al., 2003). Also, tapering of corticosteroids, which are known CYP3A4 inducers (Sewing, 1994), would lead to a decrease in the enzyme induction and subsequent higher drug exposure.

In the present study, of 56 patients who did not have HTN at baseline, 44 (78.6%) had developed HTN after being on TAC during the follow-up period. HTN after KT has been associated with earlier graft failure and higher mortality of the recipient (Farouk and Rein, 2020). However, the multivariate logistic regression analysis revealed a nonsignificant association between high TAC C_0_/D ratio or TAC C_0_ levels and the development of HTN, which is in agreement with previous studies (Staatz and Tett, 2004). However, age ≥40 years and male gender were associated with the development of HTN in our cohort. Previous studies did not identify factors associated with TAC-induced HTN rather focused on its mechanism (Staatz and Tett, 2004; Farouk and Rein, 2020).

The current results showed that from the 196 nondiabetic patients at baseline, 69 (35.2%) had developed PTDM after being on TAC during the follow-up period. PTDM is associated with poorer graft and patient survival as well as increased incidence of infections and cardiovascular events (Davidson et al., 2003). Previous studies reported PTDM incidence rates of 2–53% (Davidson Wilkinson, et al., 2003). The reported prevalence of PTDM in Kuwait during the period from 1989 to 1998 was 27.5% (Johny et al., 2002), which is lower than the present finding. This might be because during that time, TAC was not introduced in the clinical practice. The DIRECT study confirmed the increased diabetogenicity of TAC (33.6%) compared with cyclosporine (26%) in a randomized controlled trial among KT recipients during the first year of PT (Vincenti et al., 2007), which is slightly lower than the present finding. The multivariate analysis revealed that PTDM was significantly more common among patients aged ≥40 years, those with high C_0_/D ratio, and obese patients. These results are in agreement with previous findings that older age and high initial TAC blood levels were the main risk factors for PTDM (Rodrigo et al., 2005). Higher TAC level was consistently reported as a risk factor for developing PTDM in previous studies (Rodrigo et al., 2005; Boloori et al., 2015; Choudhury et al., 2020). Several IS protocols have been proposed to reduce the incidence of PTDM, including early steroid or calcineurin inhibitor (CNI) withdrawal (Kasiske et al., 2000; Pirsch et al., 2015; Van Sandwijk et al., 2018). Unfortunately, the results of early steroid or CNI withdrawal from IS regimens have demonstrated an increased rate of acute rejection and have not consistently achieved better renal function (Kasiske et al., 2000; Desai et al., 2017). Therefore, it is important to consider risk vs. benefit to balance the risk of developing PTDM vs. rejection. The current result that PTDM was significantly more common among obese patients is in consistence with previous findings (Davidson et al., 2003; Al-Ghareeb et al., 2012; Abdulrahman et al., 2018).

CNIs are known to be associated with dyslipidemia by reducing Low-density lipoprotein – Cholesterol through interfering with the binding of LDL-C to its receptor and interfering with bile acid synthesis, which leads to LDL receptor downregulation (Agarwal and Prasad, 2016). The present findings showed that 51.9% of the patients who did not have dyslipidemia at baseline developed dyslipidemia after being on TAC during the follow-up period. Overweight, obesity, and age ≥40 years were found to be significantly associated with the initiation of antilipidemic medications. This is in agreement with a previous study that reported age and BMI as risk factors for developing new-onset dyslipidemia among KT recipients (Borda et al., 2011). Moreover, patients with a higher TAC C_0_ showed a lower risk of developing dyslipidemia in the present study. The result might be related to being TAC, causing less dyslipidemia than CsA (Staatz and Tett, 2004; Borda et al., 2011). However, the aim of the current study was not to do head-to-head comparison with CsA to draw definite conclusions.

One of the factors that deserve some attention is the absence of association between concomitant medications that increase TAC concentration and the development of TAC SEs in our cohorts. In the present study, more than 90% of patients were on PPIs. Interaction between PPI and TAC had been reported to increase TAC C_0_, where frequent dose modifications were needed (Vavic et al., 2014). However, the clinical significance has not yet been studied. The results from the present study showed that the interaction had no significant clinical impact on the development of TAC SEs, and the results might change with other concomitant medications.

In the present study, the impact of C_0_/D ratio was stronger than the absolute C_0_, which suggest that C_0_ alone might be a weak predictor for TAC clinical outcome. In a recent consensus guideline for TAC TDM, TAC AUC was considered the best PK parameter associated with clinical effects (Brunet et al., 2019). However, TAC C_0_ is mostly used in transplant centers because it is easy to measure and interpret. Of note, the correlation between C_0_ and AUC is poor and translates into variable AUC/C_0_ ratios, which means that patients with identical C_0_ may have very different AUC_0–12_. However, no prospective studies of clinical outcomes have been published in adult and pediatric transplant recipients to assess the potential benefits of AUC_0–12_ monitoring compared with C_0_-monitoring approach. Therefore, whole-blood C_0_ is still used to adjust TAC dosing in most transplant centers until more evidence emerges.

The results from the present study indicate the need for interventions for the low physicians’ adherence to the local hospital protocol. There is a lack of literature in studies to determine the level of physicians’ adherence to the KT protocols, identify reasons for low adherence, and evaluate interventions to improve their adherence to these protocols. Possible suggested interventions to improve physicians’ adherence to protocols are clinical pathways that comprise an order sheet with patient care goals to provide the sequence and timing of actions necessary to achieve goals, and the multidisciplinary teams, including pharmacists, that are found to be consistently successful in increasing prescription of target dose medications. Pharmacists were found to be of added value to the transplant team, and evidence supports their positive impact in the care of transplant recipients (Sam et al., 2018). Future research should explore the genetic polymorphisms distribution among our patients followed by revisiting the local hospital protocol to assess whether genetic-based or weight-based dosing protocol would be appropriate. Also, the determination of TAC pattern of use with the rejection rate in our population would be of added value because it was not assessed in the present study. Moreover, the reasons behind clinicians’ nonadherence to protocol should be explored to facilitate the design of future targeted interventions.

### Strengths and Limitations

The strengths of the current study include i) the use of appropriate sample size to generate a representative data about the study population and ii) the use of real-world data derived from the day-to-day clinical practice in terms of dosing and follow-up of KT recipients.

There were certain limitations in the present study, which include i) the retrospective nature of the study and that the findings should be confirmed with a prospective assessment particularly for the SEs; ii) the lack of CYP3A5 genotyping in the collected data, which has been recently recommended for initial TAC dose calculation (Brunet et al., 2019); iii) the immunoassay method used for the assessment of TAC level in this study, which is considered inferior to more specific liquid chromatography with mass spectrometric detection (Brunet et al., 2019); iv) lack of identifying the physicians’ reasons for low adherence to the protocol, and v) lack of assessing patients’ adherence to the use of TAC as a possible source of variabilities and low achievement of therapeutic targets; vi) the absence of a definite differentiation between TAC SEs and clinical conditions caused by other etiologies, and vii) lack of assessment of correlation between rejection rate and TAC concentration.

## Conclusion

The current study showed that less than two-fifths and about two-thirds of patients achieved target TAC C_0_ levels during the first month of PT and by week 52, respectively. High TAC C_0_/D ratio, age ≥40 years, male gender, and BMI ≥25 kg/m^2^ were associated with the occurrence of SEs. These findings warrant efforts to implement targeted multifaceted interventions to improve TAC prescribing and monitoring after KT.

## Data Availability Statement

The raw data supporting the conclusions of this article will be made available by the corresponding author on reasonable request.

## Author Contributions

SA and AA conceived the research design. SA, AAA, and OG were involved in data acquisition; SA, AAA, MS, and AK analyzed the data and had full access to the data; SA and MS drafted the original version of the manuscript; all authors participated in the review and editing of the manuscript.

## Conflict of Interest

The authors declare that the research was conducted in the absence of any commercial or financial relationships that could be construed as a potential conflict of interest.
