# Supplementary material for: Monitoring Tacrolimus Trough Concentrations During the First Year After Kidney Transplantation: A National Retrospective Cohort Study
Source: Front Pharmacol. 2020 Nov 20;11:566638. doi: 10.3389/fphar.2020.566638 (PMC7919378; doi:10.3389/fphar.2020.566638)
Supplement: Supplementary file 1 [file Table1.docx]

**Table S1.** Univariate analysis of factors associated with TAC side effects using whole blood tacrolimus concentration

| **Characteristics** | **Developed hypertension after initiation of TAC and on antihypertensive medication(s)**  **(n=44)** | | | **Developed diabetes after initiation of TAC and on antidiabetic medication(s)**  **(n=69)** | | | **Developed dyslipidemia after initiation of TAC and on antilipidemic medication(s)**  **(n=121)** | | |
| --- | --- | --- | --- | --- | --- | --- | --- | --- | --- |
|  | **OR [95% CI]** | | **P-value** | **OR [95% CI]** | | **P-value** | **OR [95% CI]** | | **P-value** |
| **Age (Years )** | | | | | | | | | |
| 18-39 | Reference | | | Reference | | | Reference | | |
| 40-59 | 1.70 | [1.14,2.54] | 0.01 | 2.17 | [1.66,2.84] | 0.00 | 2.02 | [1.66,2.47] | 0.00 |
| ≥ 60 | 2.37 | [1.16,4.85] | 0.02 | 2.45 | [1.65,3.63] | 0.00 | 3.21 | [2.44,4.23] | 0.00 |
| **Gender** | | | | | | | | | |
| Male | Reference | | | Reference | | | Reference | | |
| Female | 0.57 | [0.39,0.84] | 0.00 | 1.43 | [1.12,1.82] | 0.00 | 1.07 | [0.90,1.28] | 0.43 |
| **BMI (Kg/m^2^)** | | | | | | | | | |
| Underweight / Normal | Reference | | | Reference | | | Reference | | |
| Overweight / Obese | 0.68 | [0.46,1.00] | 0.05 | 2.21 | [1.69,2.89] | 0.00 | 1.43 | [1.19,1.72] | 0.00 |
| **TAC C_0Wb_/D Ratio [ng/ml]/mg** | | | | | | | | | |
| <1 | Reference | | | Reference | | | Reference | | |
| 1-2 | 1.13 | [0.70,1.84] | 0.61 | 1.66 | [1.17,2.35] | 0.00 | 0.95 | [0.75,1.19] | 0.64 |
| >2 | 0.78 | [0.49,1.23] | 0.28 | 2.03 | [1.45,2.86] | 0.00 | 1.13 | [0.90,1.42] | 0.28 |
| **TAC C_0Wb_ [ng/ml]** |  |  |  |  |  |  |  |  |  |
| <8 | Reference |  |  | Reference |  |  | Reference |  |  |
| 8-11 | 0.87 | [0.55,1.37] | 0.55 | 1.08 | [0.81,1.45] | 0.59 | 0.68 | [0.55,0.84] | 0.00 |
| >11 | 1.44 | [0.90,2.29] | 0.12 | 0.83 | [0.62,1.12] | 0.22 | 0.62 | [0.50,0.77] | 0.00 |
| **Use of medications that increase TAC level** | | | | | | | | | |
| No | Reference | | | Reference | | | Reference | | |
| Yes | 0.94 | [0.64,1.39] | 0.77 | 1.26 | [0.98,1.62] | 0.07 | 0.91 | [0.76,1.09] | 0.30 |

^OR: odds ratios, CI: confidence intervals, TAC: Tacrolimus, C^_0_^/D: concentration/dose, BMI: Body mass index.^
